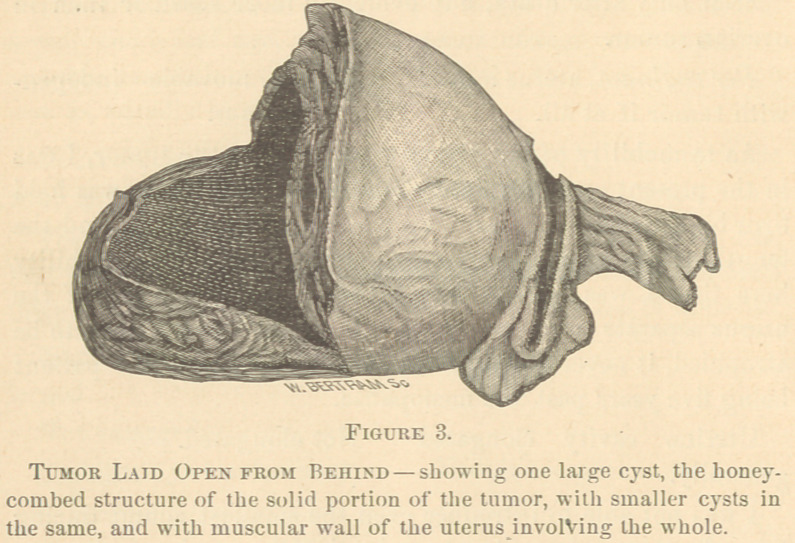# A Case of Uterine Fibro-Cystoma

**Published:** 1877-02

**Authors:** Franklin Staples

**Affiliations:** Winona, Minn.


### EXAMINER.

Vol. XXXIV.—FEBURARY, 1877.—No. 2.

### Original Communications.

### A CASE OF UTERINE FIBRO-CYSTOMA.

By FRANKLIN STAPLES, M.D., Winona, Minn.

Mrs. B., aged 51, died in Winona, Minn., Aug. 31, 1876, from what was supposed to be ovarian tumor; but what was found, on *post mortem* examination, to be uterine fibro-cystoma.

I was called to attend the case only a few days before death. There was extreme emaciation, complete loss of appetite, with diarrhœa and an aphthous condition of the mucous membrane of the mouth. Death resulted from septicæmia and prostration.

Dr. J. B. McGaughey, of Winona, assisted me in the autopsy. On making the incision through the attenuated abdominal walls, we came upon what we supposed was a large ovarian cyst, occupying, equally on each side, the whole abdominal cavity, crowding the intestines upward and backward. There was no fluid in the peritoneal cavity. On feeling for the pedicle we found, in the inguinal region of both sides, attachments which proved to be from the broad and round ligaments of the uterus; and in front and beneath what we at first supposed to be visceral and parietal adhesions, but what proved to be simply the reflections of the peritoneum to the bladder and pelvic parietes. In short, we had, instead of an ovarian cyst, not simply a subserous fibroid attached to the fundus of the uterus, but a fluctuating tumor involving the uterus itself; the broad ligaments with their inclosures extending from the fundus of the mass. The whole was easily removed by dividing the attachments to the inguinal regions, the peritoneum at its reflections from the tumor and the vagina just below the os uteri. The weight of the mass was 14½ pounds.

The appearance of the tumor: 1st, On its anterior aspect and without disection. 2d, On the left side with the uterine cavity laid open; and, 3d, With the mass laid open from behind showing the cavity of the large cyst, is shown in the cuts 1, 2 and 3.

FIGURE 1.

ANTERIOR ASPECT OF TUMOR — showing uterine appendages in their relation to the Mass, and the os and cervix uteri.

The interest in this case is not alone on account of the rarity of this form of tumor, but especially because of the liability of confounding, in diagnosis, this with ovarian cystoma.

The rules laid down by Wells, as a guide in the differential diagnosis between uterine and ovarian tumors, do not hold in many particulars when the uterine tumor is of the cystic variety, and especially when in case of a monocyst of large size.

The following points from what of the history of the case I was able to obtain, and from examination before and after death, are given in connection with Peaslee's table of differential diagnosis between uterine fibro-cystoma and ovarian cysts.

FIGURE 2.

LATERAL VIEW OF TUMOR — with the uterine cavity laid open, showing the tumor to be in the posterior uterine wall.

| UTERINE FIBRO-CYST. 3d stage. | OVARIAN CYST. 3d stage. |
| --- | --- |
| Occurs after thirty almost always. | Occurs earlier than thirty years, as well as later. |
| My patient was 51 years old. | |
| Slow growth at first. Rare. | More rapid, and more common. |

Ten years ago complained of difficulty in lower left side of abdomen. She was fleshy and no distinct tumor was made out. There was lameness through the left hip. Her general health had always been good, but from early life she had complained of pain in the back.

| | |
|---|---|
| Expression good till very large. | Expression characteristic (*Facies ovariana*). |

There was the peculiar expression and emaciation of the face which Wells, Brown and Peaslee have described as charteristic of the subject of ovarian tumor in the advanced stage.

| | |
|---|---|
| Complexion dark and injected, (*facies uterina.*) Sometime florid. No emaciation. | Pale, emaciated. |

The first description would apply to the patient until about a year and a half ago, when a gradual change was apparent; and for several months before her death the last description was as equally applicable.

| | |
|---|---|
| General health for a long time good. | Has failed by the end of the second stage. |

The general health was good until near the end of life. For the last two years, however, the stomach has been irritable sometimes, and there has been occasional loss of appetite.

| | |
|---|---|
| Abdominal veins not enlarged. | Enlarged. |

Not enlarged.

| | |
|---|---|
| Umbilicus not prominent. | Umbilicus prominent. |

Somewhat prominent.

| | |
|---|---|
| No amenorrhœa. Menorrhagia seldom. | Amenorrhœa. |

Menstruation was regular and normal until five years ago. No menstruation since. Her age was 51. She was never pregnant, had been married 34 years.

| | |
|---|---|
| Kidneys normal, | Kidneys inactive. |

Variable; generally normal; sometimes inactive.

| | |
|---|---|
| Tenderness on pressure at first. | Not tender. |

There may have been tenderness at first, but not in the later history.

| | |
|---|---|
| Elasticity, afterward evident fluctuation. | Fluctuation throughout its course. |

Only examined late in its course; then very decided fluctuation.

| | |
|---|---|
| Surface lobulated at first. May remain so. | Not lobulated, except in polycysts. |

Not lobulated.

| | |
|---|---|
| Cyst wall of livid hue. Very vascular. | Cyst wall of lighter color. Less vascular. |

Seen only after death, but evidently more vascular than in ovarian tumor.

| | |
|---|---|
| *Per vaginam*, uterus moved with tumor if at all. | Uterus movable independent of tumor. |

As to mobility of the uterus separate from the tumor, I was in the present case completely mistaken. The tumor was held firm by the abdominal parietes closely drawn over it; and the length of the os and cervix (see cuts) allowed such mobility here that I was led to believe that I had the whole of the uterus separate from the tumor. I believed the uterus to be atrophied, it never having been impregnated, and the patient being five years past the menopause.

| | |
|---|---|
| Uterine cavity elongated generally. | Not elongated. |

I was not able to introduce even the smallest sound during life. In the *post mortem* examination the uterine cavity was found to be elongated over half the circumference of the tumor. (See cut No. 2.)

| | |
|---|---|
| Fluid, yellow, serous, with little albumen, or fibrinous like lymph, and spontaneously coagulable. But it may be dark brown or hæmorrhagic. | Lighter in monocysts not before tapped; highly albuminous; sometimes colloid. |

There was in the large cyst four pints and a half of dark hæmorrhagic, serous fluid. It was without odor. There was in the cavities and interstices of the solid portion of the tumor considerable sero-purulent matter, of a light muddy color. The structure of this part of the mass had evidently suffered change, had been softened and partially broken down by recent changes tending to suppuration. This accounts for the septicæmia which was the immediate cause of death.

Concerning this form of tumor Dr. Peaslee has the following: "If we follow the development of the uterine fibroma, we find that cysts are liable to be developed in it as a secondary formation. We then have one of the forms of fibro-cystic tumor of the uterus.

FIGURE 3.

TUMOR LAID OPEN FROM BEHIND—showing one large cyst, the honey-combed structure of the solid portion of the tumor, with smaller cysts in the same, and with muscular wall of the uterus involving the whole.

"Recognized as a distinct class of tumors but a few years since, and first described by Cruveilhier, they have frequently been mistaken for ovarian cysts by the ovariotomists. Sometimes they have been removed as ovarian cysts and the mistake discovered only by a subsequent examination of the mass removed, or by a *post mortem* investigation which showed both ovaries to be in a normal condition."

I. Baker Brown remarks that he knows of no distinguishing signs between uterine fibro-cyst and ovarian cystoma, and Dr.

Peaslee has himself operated for the removal of an ovarian oligocyst in a case of a uterine fibro-cyst, and this even after a tapping by which he removed from the principal cyst 40 pounds of a brownish fluid. This, however, was in the case of a sub-serous cyst attached to the fundus uteri by a pedicle an inch in diameter and a quarter of an inch long. The mass weighed 50 pounds. It was successfully removed, and recovery took place.

More nearly allied to the present case was that reported in the *Boston Medical and Surgical Journal*, for July 13, 1876, as occurring in the practice of Dr. Gilman Kimball, of Lowell, Mass. The tumor was described as a fibrous growth extending by prolongation to the uterus and thoroughly blended with that organ, making one continuous substance. After removal the mass was found to consist of 20 pounds of straw colored fluid and five pounds of solid material; the latter consisting of the cyst, the fibrous growth, both ovaries, parts of the suspensory ligaments of the uterus and the uterus itself. Recovery followed.

Dr. Wm. T. Lusk, in December, 1875, presented a specimen and reported a case of pregnancy with fibro-cystic tumor of the uterus, in which there was almost complete occlusion of the cervix uteri. In delivery ecclampsia occurred—version—post partum hæmorrhage, and death from peritonitis and collapse. *Post mortem*, the cavity of the uterus was found to occupy the right side of the growth. The tumor was of the fibro-cystic variety. It was honey-combed, with cells containing a clear fluid. It weighed six and a half pounds. There were extensive adhesions with the intestines. (*Am. Jour. of Obst.*, April, 1876.)

Concerning the comparative frequency of fibro-cystic disease of the uterus, we have the following from eminent writers:

Peaslee. "Fibro-cystic tumors of the uterus were first distinctly recognized only very recently, and are quite rare. Only 14 cases had been reported in 1869, and two of these were detected after death by Kiwisch and Cruveilhier. They may be either sub-peritoneal or interstitial."

"Cruveilhier thinks this form of fibro-cyst is produced by the progressive dilatation of lymphatic vessels; and states that they are always consecutive to some mechanical obstacles to the circulation of the lymph."

"Klob merely quotes a single case from Kiwisch, and alludes to one described by Cruveilhier, after designating such instances as very rare and extraordinary."

T. Spencer Wells. "Of the first 130 operations for tumors (115 being ovariotomy), only two cases proved to be fibro-cystic tumor of the uterus."

Dr. Keith. "In 100 operations of ovariotomy only one case of uterine fibro-cyst occurred."

Dr. Peaslee, from his own observation, and the reports of Drs. T. A. Emmet, Charles Clay and C. C. Lee, concludes that uterine fibro-cysts are far more common in this country than in Europe.

In the "Report on the Progress of Gynæcology during the year 1875," in *Am. Jour. Obstetrics* for April, 1876, is a summary of recent literature and operations for large fibroids and fibro-cystic tumors of the uterus by gastro-hysterotomy. The operators were Thomas Keith, Edinburgh; C. H. F. Routh, London; Jas. R. Chadwich, Boston; W. G. Drake, Atlanta, Ga.; Theodore Billroth, of Vienna; E. Bockel, Robt. Barnes, London; and E. R. Peaslee and T. G. Thomas, of New York. Of eleven operations reported five resulted in recovery.

## Figures and Tables

**Figure 1. f1:**
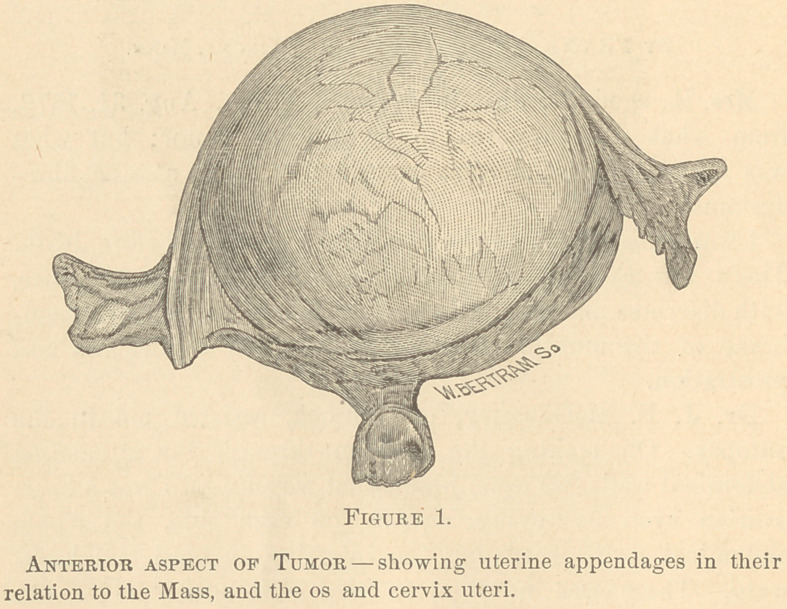


**Figure 2. f2:**
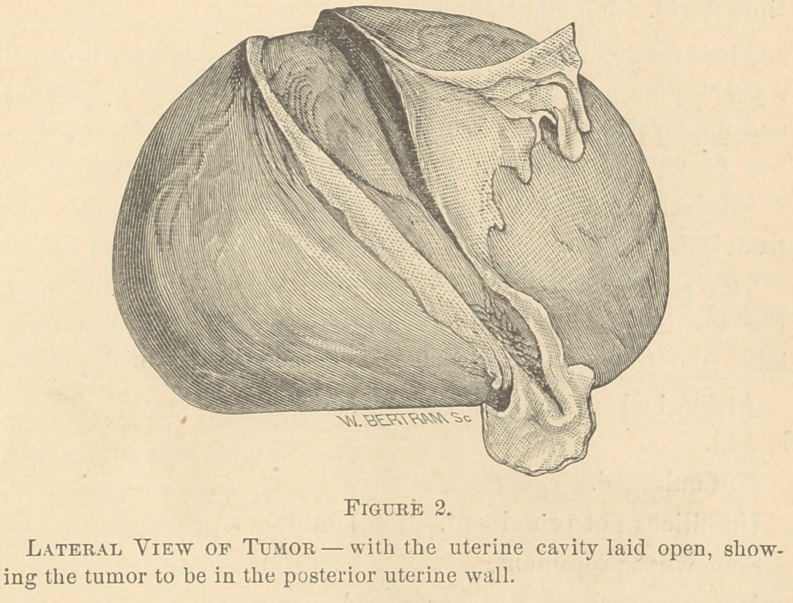


**Figure 3. f3:**